# The Impact of COVID-19 Instigated Changes on Loneliness of Teachers and Motivation–Engagement of Students: A Psychological Analysis of Education Sector

**DOI:** 10.3389/fpsyg.2021.765180

**Published:** 2021-10-12

**Authors:** Abir El Telyani, Panteha Farmanesh, Pouya Zargar

**Affiliations:** Faculty of Business and Economics, Department of Business management, Girne American University, Karmi, Cyprus

**Keywords:** COVID-19, educational psychology, motivation, engagement, university lecturers, learning theories

## Abstract

Upon the spread of the global pandemic of COVID-19, education was transformed online in an abrupt manner. Amid this change, the education sector did not have room for proper decision-making and understanding of psychological effects. This theoretical analysis aims to contribute to the proposed Frontiers Research Topic, through (a) in-depth analysis of the pandemic status and behavioral psychology and (b) examining educational psychology from the perspective of teachers regarding sudden changes. As a result, implications are suggested based on interviews, linking to extant literature. The current research recognizes the difference between online learning and emergency remote education. While the former comprises prepared means of teaching and assessment, the latter is unaccompanied by such preparedness. Thus, there are variations in the outcomes of learning, motivation, and engagement. Scholars, teachers, deans, and educational managers can benefit from current results.

## Introduction

With the pandemic of COVID-19 surfaced, its effects were unanimously researched by experts across the world. It has been the core concern of scholars in particular (e.g., Leung et al., 2020; WHO Regional Office for Europe, 2020; de la Fuente et al., 2021). As the occurrence of the pandemic is unprecedented, it is vital that its psychological impacts are examined and understood to provide strategies for better adjusting to the current status quo and aim for resilience for future pandemic-like situations. The current research looks into the psychological effects of the COVID-19 pandemic on undergraduate students from the perspective of teachers. Motivation, engagement, and learning outcomes (e.g., success) are included in this study. Educational psychology can be defined as a subgroup of psychology that specifically addresses education (de la Fuente et al., 2021). Educational psychology entails various aspects, such as the effects of COVID-19 on the development at certain stages (physical, socio-moral, motor, cognitive, and linguistic) (Liu et al., 2020), learning (Cachón-Zagalaz et al., 2020), stress caused by changes for students, teachers, and parents (Valadez et al., 2020), changes in educational contexts, and achievement (Martarelli et al., 2021), and rise of technological means (Obrero-Gaitán et al., 2020).

Within the scope of current research, the education sector and universities specifically were forced to switch traditional in-person classes to online systems. This emergency remote educational format of teaching undeniably has had significant psychological impacts on both teachers and students. This is while online education platforms have been in place for several years, where individuals can take an array of courses and obtain certifications. In this sense, teachers had little-to-no time to prepare for the pandemic to completely change their teaching styles considering learning, motivation, engagement, and educational outcomes of students (Chacón-Fuertes et al., 2020; Daniels et al., 2021). It has been noted in recent studies that the format of education will continue to be online regardless of the motivation for returning to in-person classes (Hyslop, 2020). Notably, both students and teachers are keen to return to the “previous” state. This research investigates the effects of the pandemic (shift to online learning in a sudden manner) on the engagement of students and motivation through the perspective of teachers.

## Literature Review

In the context of current research, Achievement Goal Theory (AGT) has been found to be appropriate, when examining the *motivation* of students (Senko and Dawson, 2017). Achievement goal theory is used to theoretically explain changes in attitudes (motivation) of teachers and students in the sudden shift of education format during Covid-19 pandemic (Daumiller et al., 2021). There are two aspects in this regard that are incorporated within this theory, namely, competence and valence. Competence is referred to as the exhibition of the level of competence of an individual compared to peers (performance and mastery goals), which are linked to desirable outcomes from the learning process (e.g., emotions, interest, regulation of self, and strategies of learning). Valence addresses the difference between approach and avoidance of motivation (Elliot, 1999). Behaviors that are the predictors of positive learning outcomes (i.e., learning new subjects) are referred to as approach motivation, while avoidance motivation is a negative form, such as failure (Huang, 2012; Daniels et al., 2021).

The *engagement* of students has been defined as a multidimensional factor that consists of various operations. In this sense, behavior, emotion, and cognition are key aspects (Fredricks et al., 2004). Behavioral engagement incorporates actions, effort, consistency, participation, and positivity within a course (Daniels et al., 2016). Feelings and/or attitudes toward a specific course are referred to as emotional engagement (affective) (Appleton et al., 2008). These feelings can vary from not being interested in high levels of interest in the course. Furthermore, cognitive engagement includes the processes of thought for students regarding a course and its task in an internal manner. Self-directed questions, additional resource findings, and discussion on course material with others are among the factors of cognitive engagement (Daniels et al., 2021). Problem solving, coping strategies, and willingness to learn are also among the dimensions of the cognitive engagement process (Appleton et al., 2008).

It is important to note that other constructs, such as learning, knowledge, skills, and satisfaction, are also vital for the achievements of students (Goegan and Daniels, 2019), especially through information and communication technologies (ICT). As the pandemic shifted the grading system of schools into CR/NCR, studies mentioned that the stress level was reduced as mere traditional factors of success were influenced. Accordingly, the perspective of the success of students has changed, which was observable by teachers during classes. The existence of the positive impact of approach goals on objective and subjective success in academia has been noted in the extant literature (Huang, 2012).

Online learning is special in its features and has its unique methods of instruction, philosophy, and subsequent psychological impacts. According to Korkmaz (2019), online learning is embedded within the premise of Reconstructionism and humanism and is linked to connectivism (Jung, 2019). The objective of online learning is to provide opportunities for learning in an equal manner and to overcome barriers (Korkmaz and Toraman, 2020). In the era of digital advancements, the learning theory of connectivism is explained through globalization, long-term learning, technology, and digital information. It has been noted that various theories are involved in this context that are chaos, network, complexity, and self-organization (Siemens, 2004). These are followed by a framework that can be categorized into different aspects, such as, diversity in opinion establishing learning and knowledge, information sources and their connections shaping learning process, non-human appliances can include learning process, the capacity of learning new things is more significant than existing knowledge of an individual, creation, and maintenance of networks and connections are crucial if learning is to be continuous, the capability of linking different concepts, notions and disciplines, the extent of which knowledge is up-to-date, and learning through the process of decision-making. What is learned and assumed to be correct today may turn to be false in the future due to changes within data. This is an important aspect of learning as it entails the notion of science and scientific learning (Tyson, 2019). In the context of connectivism, it is set that participation is imperative for learning so that teachers can deliver knowledge to students through interactions (Bozkurt, 2014). Collaboration, connectivity, emphasis on students, community, exploration, knowledge sharing, authenticity, and experiences are among the characteristics of online learning from the nexus of educational psychology and learning theories (Mayer, 2019; Weidlich and Bastiaens, 2019). Social, cognitive, and teaching presence alongside communities of online learning and learners who seek to learn internally are also noted in this context (Su, 2016).

Current study takes the aforementioned factors into consideration based on the awareness of teachers and decision-makers upon occurrence of Covid-19 pandemic as changes in education system were abrupt and mandatory. Thus, the perspective of the teachers regarding the engagement of students and motivation is looked into, while the notion of teachers' working in a solidary format (*workplace loneliness*) is assessed. Through understanding these factors and how they have been influenced by the COVID-19 pandemic, implications for the era after the pandemic can be drawn to assist educators. The use of resources digitally, equipment, internet access, platforms, familiarity, and devices were noted as key challenges for educators (Huber and Helm, 2020; Quezada et al., 2020; UNESCO, 2020). According to Wang et al. (2021), ineffective communication, work-home interference, and workplace loneliness are noted as major challenges for teachers during the COVID-19 pandemic. In the context of current research, workplace loneliness is the situation in which teachers have little-to-no interactions with their colleagues and/or supervisors. This was combined with other restrictions, such as not being able to have social gatherings and meet friends, which further caused loneliness. This factor has vivid effects on well-being and performance at the job (Wang et al., 2021).

Loneliness is defined as a sense of being empty, alienated, and having no particular relationship and linkage with others (Dor-Haim and Oplatka, 2021). It is associated with various negative psychological and organizational outcomes, such as decreased performance, lesser work quality, lowered motivation and commitment, diminished job satisfaction, increased turnover intentions, and reduced well-being (Cacioppo and Cacioppo, 2014; Ozcelik and Barsade, 2018). While the concept of loneliness has been examined in the education sector, this factor and its effect on university teachers after the occurrence of the pandemic have not been overly discussed. Hence, the current research looks into this factor from the perspective of teachers to provide a better understanding of how COVID-19 consequences impacted the well-being and performance of teachers due to perceived loneliness at work during emergency remote education. Studies conducted in the same context have reported negative impacts of loneliness on performance, engagement, job satisfaction, well-being, and functioning (Bakır and Aslan, 2017; Dor-Haim and Oplatka, 2021). This element is addressed in the current research to see if the loneliness of teachers is influential regarding what they perceive of engagement and motivation of students.

Motivation is complex by nature and has been studied extensively in the field of psychology and other relevant disciplines. It impacts behaviors, thought processes, and the duration of time individuals dedicate to their tasks (Urdan and Schoenfelder, 2006). In the field of academia, motivation for students implies a continuation of learning and combined joy (Zimmerman, 2008). This is while lack of motivation leads to failure in the academic field. Various factors can impact the motivation of learners (e.g., teachers' approach and attitude, expectations, family, social values), which have direct effects on the performance and participation of students in classes. Social cognitive theory (SCT) and self-determination theory (SDT) are used as motivational theories (Bandura, 1989). SCT is also referred to as social learning theory (SLT), which includes the social context of reciprocation, interaction, and learning for an individual in given environments and performed actions. Social reinforcement both externally and internally is within SCT, with added emotions and cognitive abilities to SLT. In other words, the actions, feelings, and thoughts of an individual impact other in the given social setting (Oden et al., 2019). Teacher interaction, student expectations, and descriptions of learning quality are among the social-contextual factors impacting ones' cognition in academia (Bandura, 1989). Classroom and school environments and individual interactions with these settings shape the motivation of a student. Autonomous motivation, controlled motivation, and a-motivation are noted in SDT (Ackerman, 2020) that lead to the achievements of students. Social elements can thus increase or diminish motivation as a psychological factor. Autonomous motivation entails intrinsic and extrinsic actions, and the importance of self-controlled motivation includes external regulation and initial regulation (reward, punishment, desire for acceptance, avoiding guilt, self-esteem, and self-involvement conditionally). A-motivation addresses the lack of intention or motivation, which varies from other forms (Rahiem, 2021).

## Methodology

This research is conducted in the business faculty and psychology department of a university in Cyprus, located in Kyrenia. Complying with ethical means of research conduct, no names or direct information is provided as per the agreement with the school and teachers. Teachers of the faculty have been systematically sampled (systematic sampling), which then according to their respective schedules were interviewed. As the current research uses several theories to address the topic at hand, the inductive approach has been deemed most appropriate. The qualitative approach was used for the collection of data and analyses (Creswell and Poth, 2017; Dor-Haim and Oplatka, 2021). This is due to the need for knowledge regarding the perspectives of the teachers on loneliness during the pandemic and how it affected the engagement and motivation of students. This approach is used to unravel the depth of the aforementioned effects in the specific context of this research (Denzin and Lincoln, 2018). It is important to note that a limitation is pinpointed as data are collected from a single faculty.

## Sampling and Data Collection

Several 20 faculty teachers were included in the data collection process through semi-structured interviews. The research used two criteria to select samples from the faculty (a) willingness to participate in the interview, (b) experienced loneliness during the emergency remote education, and (c) had over 100 students per semester. Information of participants is presented in Table 1. Semi-structured interviews were conducted through Zoom, complying with the rules and regulations during the COVID-19 pandemic. This approach ensures a systematic understanding of the case at hand through repeated questions from all the participants (Marshal and Rossman, 2016). Interviews were held by the first and second authors throughout the spring semester from March 2021 to June 2021. Participants were permitted to record the interviews for further transcription. Moreover, interviews were conducted anonymously, and obtained string data were deleted alongside recordings upon the completion of coding the process (keywords and their synonyms were coded through MAXQDA, v2020, VERBI Software, Berlin).

**Table 1:** Participants' profile.

**Factor**	**Count**	**Total**
Age range	Min 30/Max 59	20
Experience	Min 2/Max 25	20
Gender	Female 13/Male 7	20
Number of students during pandemic classes (approx.)	Min 145/Max 220	+3,000

All authors are active in the field of psychology and education and took the responsibility for different aspects of the research. Furthermore, all authors combined their interpretations to reach conclusions. The third author evaluated the findings and noted varying aspects of the results. Interviews were conducted in three stages, namely, the initial stage (questions regarding loneliness at work during the pandemic), the second stage (perspective of the teachers on engagement and motivation of students), and the final stage (where participants provided any further remarks on the subject). The interviewer took social desirability into account and thus, no personal perception, judgment, opinions, and reactions were exhibited throughout the session. Collected data were then organized, categorized, and coded for patterns, tested, and interpreted (Marshal and Rossman, 2016). Themes were created based on codes to highlight keywords in the context of this research. Responses were compared and texts paraphrased to fit the current narrative (Strauss and Corbin, 1998).

## Results and Discussion

The analysis took several factors into account that are reported below. While demographic information is presented in Table 1, and Table 2 exhibits the factor/keywords selected in the thematic analysis and their categories. The percentage extracted value shown in Table 2 is based on the number of repetitions for each category.

**Table 2:** Factor % of repetition.

**Factor/keyword**	**Categories**	**% Extracted**
Loneliness (performance, stress, physical) teachers	- Lack of energy	75%
	- Interpersonal life	34%
	- Sadness	57%
	- Frustration	54%
	- Sleep/eat issues	13%
	- Autonomy	7%
	- Added focus on personal life	7%
	- Reduced performance	75%
	- Anxiety	44%
Engagement students	- Decreased attendance	66%
	- Reduced interaction	85%
	- Not answering questions	75%
	- Not engaging in solving problems	80%
	- Engagement lowered	65%
Motivation students	- Task completion reduce	75%
	- Resource usage increased	35%
	- Online practices and assignments done	40%
	- Online groups activities increased	55%
	- Dedication/achievements enhanced	35%
	- Performance reduced	65%

### Impact of Loneliness (Caused by the Pandemic)

Table 2 shows the importance or severity of each item from the perspective of participants. As keywords and synonyms for each item are coded, repetition increases the weighing. Hence, higher percentages are derived from keywords of an item being repeated significantly. It was found that teachers noted significant effects on their level of performance and emotions (particularly stress). This is while the physical health aspect was not highlighted in their response. Interestingly, two teachers noted positive impacts of loneliness as being able to focus more on their time. While the aforementioned were outliers, other teachers noted negative effects. It was also found that some teachers had issues of internet access, platform issues, and a lack of familiarity with online tools. Linked to SDT, this lowers the sense of competence and autonomy of teachers, which negatively impacts their emotions. Furthermore, through online platforms, teachers noted linkage with SCT as they did not have the means for observing others and interact with their colleagues through this newly applied change. Teachers felt a lack of energy for their preparation and working hours. Interpersonal relationships were also noted as an influential factor on teachers' level of felt loneliness and performance-related outcomes. Working in the environment of a home was not suitable for 35% of participants (Table 2). Frustration, sadness, and changes in sleep or eating were noted in five participants, which were related to the loneliness that was caused by the COVID-19 pandemic.

Teachers stated that loneliness negatively affected their professional performance. Some reported lowered confidence levels as they did not have proper communications with their work environment and their students. Their motivation for teaching was also negatively impacted by their perceived loneliness, which was further combined with stress, nervousness, and anxiety. Many teachers noted the negative effects of loneliness on their creativity in delivering course material. Frustration regarding lack of interaction, communication, classroom engagement, and work environment was repeatedly mentioned by participants, which are linked with the theoretical framework of this study. The impacts of loneliness on physical health were not found significant in responses (e.g., overeating, insomnia, etc.).

### Engagement and Motivation of Students in Online Classes

It was significantly noted by all the participants that students had a vivid reduced engagement with the course throughout the pandemic. From large classes with over 50 students (Table 1), attendance was lowered and engagement was reduced to <10 students per session (some classes of +60 students had engagement limited to 2–4 students per session). The motivation was lowered from the perspective of teachers. This can be linked to AGT from both competence and valence dimensions. Teachers felt that students did not show a rise in involvement toward their desired subjects or tasks. Their competence was further under question as uncertainty rose regarding success or failure in the abruptly shifted system. Furthermore, the performance of students was notably diminished as the overall mean of scores decreased by ~15–20%. This is a reflection of lowered motivation according to the participants. Embedded in the premise of SCT, students had limited interactions, were forced to remain in their homes, and were experiencing sudden changes in their education and other aspects of life. Teachers noted that both engagement and motivation were decreased during the pandemic.

## Conclusion

As the COVID-19 pandemic is still ongoing, it is imperative that both teachers and students are focused on ensuring that knowledge is properly transferred to the next generations. This requires preparedness, strategy, and fast responses to such events. Crucially, psychological factors should be the focus of decision-makers in this field so that educational psychology and psychosocial contexts are taken into consideration. Consequences that will rise from lack of motivation and engagement (both teachers and students) can have direct effects on the future of education. It is also important to note that the psychological state of teachers should be addressed and their well-being noted so that they can better overcome current challenges. In this sense, the motivation and engagement of students should be regarded as behavioral, personal, and important factors. Universities must imply technologies that allow teachers to hold classes effectively until the status quo has changed. This means usage of fast internet, proper tools and equipment, and training for mastering these elements. This research highlights the collaboration between school managers, IT teams, teachers, and faculty deans/vice deans. This can lead to enhanced outcomes in the performance of teachers and subsequently, students.

## Data Availability Statement

The original contributions presented in the study are included in the article/supplementary material, further inquiries can be directed to the corresponding author.

## Ethics Statement

The studies involving human participants were reviewed and approved by Girne American University Ethical Committee. The patients/participants provided their written informed consent to participate in this study.

## Author Contributions

AT: interview and writing. PF: supervision and interview. PZ: analysis and final writing. All authors contributed to the article and approved the submitted version.

## Conflict of Interest

The authors declare that the research was conducted in the absence of any commercial or financial relationships that could be construed as a potential conflict of interest.

## Publisher's Note

All claims expressed in this article are solely those of the authors and do not necessarily represent those of their affiliated organizations, or those of the publisher, the editors and the reviewers. Any product that may be evaluated in this article, or claim that may be made by its manufacturer, is not guaranteed or endorsed by the publisher.
